# Mesenchymal stem cell homing to improve therapeutic efficacy in liver disease

**DOI:** 10.1186/s13287-022-02858-4

**Published:** 2022-05-03

**Authors:** Mengqin Yuan, Xue Hu, Lichao Yao, Yingan Jiang, Lanjuan Li

**Affiliations:** 1 Department of Infectious Diseases, Renmin Hospital of Wuhan University, Wuhan, China; 2 State Key Laboratory for Diagnosis and Treatment of Infectious Diseases, National Clinical Research Center for Infectious Diseases, Collaborative Innovation Center for Diagnosis and Treatment of Infectious Diseases, The First Affiliated Hospital, College of Medicine, Zhejiang University, Hangzhou, China

**Keywords:** Mesenchymal stem cell (MSC), Liver diseases, MSC homing, Improvement strategies

## Abstract

Mesenchymal stem cell (MSC) transplantation, as an alternative strategy to orthotopic liver transplantation, has been evaluated for treating end-stage liver disease. Although the therapeutic mechanism of MSC transplantation remains unclear, accumulating evidence has demonstrated that MSCs can regenerate tissues and self-renew to repair the liver through differentiation into hepatocyte-like cells, immune regulation, and anti-fibrotic mechanisms. Multiple clinical trials have confirmed that MSC transplantation restores liver function and alleviates liver damage. A sufficient number of MSCs must be home to the target tissues after administration for successful application. However, inefficient homing of MSCs after systemic administration is a major limitation in MSC therapy. Here, we review the mechanisms and clinical application status of MSCs in the treatment of liver disease and comprehensively summarize the molecular mechanisms of MSC homing, and various strategies for promoting MSC homing to improve the treatment of liver disease.

## Introduction

Viral and alcoholic liver disease, drug-induced liver disease, autoimmune hepatitis, and primary biliary cirrhosis can eventually progress to end-stage liver disease, which has gradually become one of the main causes of death globally [1]. Multiple therapeutics have been developed to target end-stage liver disease, including drugs, artificial livers, and endoscopic and vascular interventions for portal hypertension. Although therapies alleviate clinical symptoms to some extent, hepatic hypofunction cannot be reversed because of the decreased number of hepatocytes [2]. Currently, orthotopic liver transplantation remains the only effective treatment for end-stage liver disease [3]. However, there are insufficient donor sources to meet clinical needs. Additionally, post-transplant rejection and high treatment costs limit their applicability [4]. Therefore, alternative treatment strategies for end-stage liver diseases are needed.

Primary hepatocyte transplantation can be used as an alternative method to liver transplantation [5]. Transplanted hepatocytes proliferate to regenerate the damaged liver and compensate for the loss of liver function. However, the practical application of hepatocyte transplantation is restricted by the availability of donor cells and their limited proliferative potential in vitro. Research on regenerative medicine and stem cells has rapidly advanced in recent years. Mesenchymal stem cells (MSCs) are pluripotent cells with self-renewal abilities that can differentiate into multiple lineages [6, 7]. Significant advances have been achieved in using MSCs to treat liver disease, both in preclinical and clinical trials conducted by scholars at home and abroad [8].

This review mainly focused on the mechanisms and clinical application status of MSCs for treating liver diseases, as well as critically discussed the process of MSC homing and the various strategies that attempt to optimize it. This article can serve as a reference for future basic and clinical research on MSCs.

## Mechanism of MSCs in treating liver disease

Mesenchymal stem cells are multi-potent stromal cells derived from the mesoderm and were first identified in the adult bone marrow in the 1970s [9]. The MSCs can be isolated from the adipose tissue, muscle, dermis, dental pulp, synovium, umbilical cord, placenta, chorionic villi, menstrual blood, breast milk, and amniotic fluid [10]. In 2006, the International Society for Cell and Gene Therapy proposed several minimal criteria for defining MSCs as follows: (1) The cells are plastic adherent when maintained under standard culture conditions; (2) the cells must express specific cell surface markers, such as CD73, CD90, and CD105; (3) the cells lack expression of typical hematopoietic markers such as CD45, CD34, CD14/CD11b, CD79a/CD19, or human leukocyte antigen–DR isotype; and (4) the cells can undergo tri-lineage differentiation into chondroblasts, adipocytes, or osteoblasts using appropriate culture media.

**Fig. 1:**
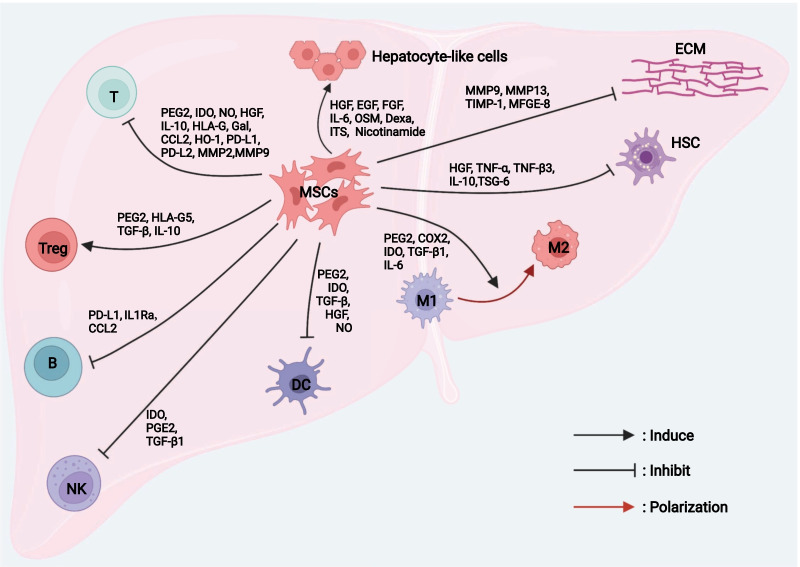
The mechanism of therapeutic effect of MSCs in liver disease. MSCs repair injured liver tissue via differentiation, immunomodulatory effects, and anti-fibrotic effects. MSCs, mesenchymal stem cells; HSC, hepatic stellate cell. *ECM* extracellular matrix, *M1* classically activated macrophage, *M2* alternatively activated macrophage, *DC* dendritic cell, *NK* natural killer cell, *B* B lymphocyte, *Treg* regulatory cell

The MSCs can be induced to terminally differentiate into multiple lineages under appropriate in vitro culture conditions. They can regenerate bone, adipocytes, endothelial cells, muscle cells, and neurons, demonstrating the potential for use in regenerative medicine [11]. MSCs are hypoimmunogenic because they lack class II major histocompatibility antigens and express low levels of class I major histocompatibility molecules. Additionally, MSCs do not express co-stimulatory molecules such as CD40, CD80, and CD86, which are important for immune recognition [12]. To clarify the active role of MSCs in treating liver disease, we briefly outline the mechanism of MSC-based therapies, as summarized in Fig. 1. And the intracellular signaling pathways in terms of MSCs regulating other cells are summarized in Table 1.

**Table 1:** Summary of the intracellular signaling pathways in terms of MSCs regulating other cells

Liver disease	MSCs source	Mechanism	Outcome	References
Liver sterile inflammatory injury	BM-MSCs	Promote Hippo signaling pathway	Shift macrophage polarization from M1 to M2 phenotype, diminish inflammatory mediators, and reduce hepatocellular damage	Li et al. [13]
Not mentioned	BM-MSCs	Inhibit CD25 expression and LKB1-AMPK-mTOR pathway	Potentiate T cell suppression	Yoo et al. [14]
Graft versus host disease	hP-MSCs	Regulate the crosstalk between Nrf2 and NF-κB signaling pathway	Inhibit the expression of PD-1 in CD4^+^ IL-10^+^ T cells, mitigate liver damage and improve redox metabolism	Zhang et al. [15]
Not mentioned	BM-MSCs	Activate Notch pathway	Increase Treg induction	Rashedi et al. [16]
Liver fibrosis	UC-MSCs	Strongly inhibit TGFβ signaling of HSCs	Inhibit HSC activation, reduce ECM deposition and liver fibrosis	An et al. [17]
Thioacetamide-induced hepatic fibrosis	BM-MSCs	Inhibit TGF-β/Smad pathway in HSCs	Reduce hepatic collagen distribution, lowered the hydroxyproline content, and rescued liver function impairment	Jang et al. [18]
Liver fibrosis	BM-MSCs	Activate Notch1 signaling pathway and inhibit PI3K/Akt pathway	Inhibit the proliferation of HSCs	Chen et al. [19]
Liver fibrosis	BM-MSCs derived exosomes	Inhibit Wnt/β-catenin pathway	Inhibit HSC activation, reduce collagen accumulation, enhance liver functionality, inhibition of inflammation, and increased hepatocyte regeneration	Rong et al. [20]

### Differentiation of MSCs into hepatocyte-like cells

Hepatocyte-like cells derived from MSCs are a promising source of cells for liver regeneration. Zhang et al. detected the expression levels of human albumin (ALB), α-fetoprotein (AFP), CK18, and CK19 in the liver tissues of CCl_4_-induced liver fibrotic/cirrhotic rats after umbilical cord (UC)-derived MSC transplantation, confirming that the transplanted cells differentiated into immature hepatocytes (epithelioid cells) and then matured into hepatocyte-like cells via a dynamic differentiation process [21]. The MSCs can differentiate into hepatocyte-like cells when incubated with growth factors or cytokines such as hepatocyte growth factor (HGF), epidermal growth factor (EGF), fibroblast growth factor (FGF), leukocyte inhibitor, IL-6, oncostatin M (OSM), dexamethasone (Dexa), nicotinamide, and insulin transferrin selenium (ITS) [22]. Can MSCs-differentiated hepatocyte-like cells serve as functional stem cells? Okura et al. found that hepatocyte-like cells differentiated from adipose-derived (AD)-MSCs exhibited the functional characteristics of hepatocytes, including the expression of ALB, secretion of urea, the activity of cytochrome P450, uptake of low-density lipoprotein, and storage of glycogen [23]. However, Campard et al. found that hepatocyte-like cells differentiated from UC-MSCs lacked various hepatic markers, such as hepotocyte paraffin 1 and hepatocyte nuclear factor 4, indicating that they did not reach the level of mature hepatocytes [24]. Notably, hepatocyte-like cells transdifferentiated from MSCs accounted for only a small fraction of the total liver volume. Therefore, more effective methods for promoting liver differentiation should be developed to enhance the efficacy of MSCs in treating liver diseases.

### Immunoregulation capacity of MSCs

Immune dysregulation in damaged liver tissue is considered the main cause of fibrosis and liver failure. Recent studies showed that MSCs exert immunoregulatory activities through intercellular contact or paracrine regulation of the congenital and adaptive immune responses.

Macrophages play a fundamental role in innate immunity. An imbalance in M1/M2 polarization is pivotal in liver injury and fibrosis. In general, MSCs tend to inhibit M1 (pro-inflammatory subtype) and induce M2 (anti-inflammatory subtype), thereby facilitating inflammation resolution and tissue regeneration. Activated MSCs promote M2-type polarization of monocytes (M0) through prostaglandin E2 (PGE2), indoleamine-2, cyclooxygenase 2 (COX2), 3-dioxygenase (IDO), TGF-β1, and IL-6 [25–27]. Li et al. showed that BM-MSCs mediate reprogramming of macrophage polarization to an anti-inflammatory M2 phenotype by promoting the Hippo signaling pathway [13]. Dendritic cells (DCs), which are the main components of the innate immune system, process antigens that they present to T cells. The MSCs inhibit the differentiation, maturation, and migration of DCs, which is mediated by soluble factors such as PGE2, IDO, HGF, TGF-β, and nitric oxide (NO) secreted by MSCs [28–30]. Natural killer (NK) cells play a key role in the front-line immune defense against invading pathogens, regulation of liver inflammation, and recruitment of circulating lymphocytes. Spaggiari et al. confirmed that MSCs can inhibit NK cells by secreting IDO and PGE2 [31]. In addition, MSCs can inhibit the expression of natural killer group 2 member D on NK cells by secreting TGF-β1 and inhibiting the cytotoxicity of NK cells in vitro, effectively reducing the levels of alanine aminotransferase and pro-inflammatory cytokines and reducing the infiltration of inflammatory cells in the liver [32].

The T and B lymphocytes are the main participants of adaptive immunity. As described above, MSCs reduce T cell activation by inhibiting DC maturation. The MSCs also significantly inhibit the proliferation of activated T cells, primarily by blocking T cells in G0/G1 phase of the cell cycle rather than by inducing T cell apoptosis [33]. Studies have suggested that the inhibitory effect of MSCs on the proliferation and activation of T cells is mediated by the secretion of a variety of soluble molecules such as NO, PGE2, IDO, HGF, IL-10, human leucocyte antigen-G (HLA-G), galectin (Gal), CC chemokine ligand 2 (CCL2), heme oxygenase-1 (HO-1), and TGF-β1 [34–37]. The PD-L1 and PD-L2 secreted by MSCs can also inhibit CD4+ T cell activation and induce irreversible T cell hyporeactivity [38]. Matrix metalloproteinases (MMPs) secreted by MSCs, particularly MMP2 and MMP9, also contribute to the inhibitory activity of MSCs by downregulating CD25 expression on the surface of responsive T cells [39]. Yoo et al. showed that MSCs suppress T cells by inhibiting CD25 translation through the LKB1-AMPK-mTOR pathway [14]. Furthermore, Zhang et al. found that human placenta (hP)-MSCs could inhibit PD-1 expression in CD4^+^IL-10^+^ T cells and alleviate liver injury in a graft versus host disease mouse model by regulating the crosstalk between Nrf2 and NF-κB signaling pathways [15]. Regulatory T cells (Tregs) play a crucial role in inhibiting immune cell-mediated hepatocyte injury during fulminant hepatitis [40]. Yan et al. found that Tregs co-cultured with MSCs induced stronger immunosuppression, which may be mediated by IL-10 secreted by MSCs [41]. Toll-like receptor-3 and receptor-4, which are highly expressed in MSCs, can induce the differentiation of Tregs through the Notch signaling pathway [16]. Furthermore, numerous soluble factors (such as PGE2, HLA-G5, and TGF-β) also play important regulatory roles in the MSCs-induced differentiation of Tregs, thereby inhibiting immune cell activation [42]. B-lymphocytes are involved in maintaining adaptive and humoral immunity by presenting antigens and acting as antibody-producing cells. MSCs can inhibit B cell differentiation, proliferation, activation, and antibody production [43]. MSCs-derived CC chemokine ligand 2 (CCL2) inhibits B cell proliferation and antibody production in B cells by inhibiting STAT3 activation and inducing paired box 5 expression [44]. The MSC-derived interleukin 1 receptor antagonist (IL1Ra) and PD-L1 inhibit the differentiation of B cells and induce the polarization of macrophages toward a M2 phenotype [45, 46].

Notably, the MSCs of different origins exhibit different immunomodulatory properties. Melief et al. found that bone marrow (BM)-MSCs and AD-MSCs showed equivalent immunophenotyping and multiple in vitro differentiation abilities. However, AD-MSCs showed higher levels of cytokine secretion (IL-6 and TGF-β1) at the same cell number. However, the reason for this difference remains unclear [47].

### Anti-fibrotic effects of MSCs

In chronic liver injury, profibrotic factors secreted by the damaged liver promote the activation and proliferation of hepatic stellate cells (HSCs), which are subsequently converted to myofibroblasts. Myofibroblasts synthesize the extracellular matrix (ECM) and release large amounts of metalloproteinase tissue inhibitor (TIMP)-1, which can reduce ECM degradation and ultimately induce ECM accumulation by inhibiting interstitial collagenase activity. In vivo and in vitro experiments showed that MSCs exerted anti-fibrotic effects mainly through paracrine signaling. The MSCs secrete a variety of soluble molecules such as HGF, TNF-α, TNF-β3, and IL-10 to inhibit HSC activation and reduce collagen production. In contrast, MSCs can directly degrade the ECM by upregulating MMPs (such as MMP9 and MMP13) and reducing the expression of TIMPs (such as TIMP-1) to reverse liver fibrosis [21]. MSCs also can inhibit HSC activation by releasing tumor necrosis factor α stimulated gene 6 (TSG-6), which can induce HSCs to transform into stem cell-like cells and improve mouse liver injury in vitro [48]. Multiple signaling pathways, such as TGF-β/Smad, PI3K/Akt, Notch, and Wnt/β-catenin, play key roles in activation of HSCs and the progression of hepatic fibrosis [49–51]. It was found that MSCs can inhibit TGF-β signaling and reduce ECM deposition and hepatic fibrosis by secreting milk fat globe-epidermal growth factor-8 (MFGE-8, an anti-fibrotic protein) [17]. Another study also shown that BM-MSCs strongly inhibited the progression of thioacetamide-induced hepatic fibrosis by suppressing TGF-β/Smad signaling pathway [18]. In addition, MSCs can directly suppress HSC proliferation via upregulating the Notch 1 expression, downregulating the PI3K/Akt or Wnt/β-catenin pathway, and thus alleviating liver fibrosis [19, 20].

## Clinical application status and challenges of MSCs in the treatment of liver disease

As described above, abundant preclinical evidence has confirmed that MSC can promote liver regeneration, which seems to be a promising method for the treatment of liver diseases. Growing evidence from clinical trials has further confirmed the effect of MSCs in treating liver diseases, particularly liver cirrhosis and acute-on-chronic liver failure (ACLF) (Table 2). Zhang et al. demonstrated that UC-MSCs significantly reduced ascites in patients with decompensated liver cirrhosis and significantly improved liver function, manifested as increased serum ALB and total bilirubin levels and decreased end-stage liver disease scores [52]. A phase I clinical trial conducted by Huang et al. showed that GXHPC1 (a cell product containing human AD-MSCs isolated and expanded from autologous donors) significantly improved liver function as well as the METAVIR score, Child–Pugh score, model for end-stage liver disease (MELD) score, and quality of life of patients with liver cirrhosis [53]. A phase II clinical trial was performed to determine the anti-fibrotic effect of BM-MSC transplantation for treating alcoholic cirrhosis [54]. The results revealed that treatment with BM-MSCs improved the Child–Pugh score and significantly reduced TGF-β1, type I collagen, and α-smooth muscle actin levels. In addition, another open-label, multicenter, randomized phase II clinical trial was conducted to assess the safety and clinical efficacy of BM-MSC transplantation in treating alcoholic cirrhosis [55]. The results showed that BM-MSCs significantly reduced the area of hepatic fibrosis and improved the Child–Pugh score. Autologous MSC infusion also showed beneficial effects on hepatic synthetic function and hepatic fibrosis in HCV-associated end-stage liver disease [56]. In addition, MSCs can also be useful for treating primary biliary cirrhosis and cirrhosis caused by autoimmune diseases [57–59].

**Table 2:** Clinical trials using MSCs to treat liver disease

Liver disease	MSCs source	Injection route	Enrolled patients	Cell doses	Follow-up	Clinical results	References
Decompensated liver cirrhosis	UC-MSCs	Vein	Control group (*n* = 15)/MSCs treatment group (*n* = 30)	0.5 × 10^6^/kg	48 weeks	Significantly reduced the volume of ascites and improvement of liver function (increased of serum albumin levels, decrease in total serum bilirubin levels, and decreased in the sodium model for end-stage liver disease scores)	Zhang et al. [52]
Liver cirrhosis	AD-MSCs	Liver	Pre-MSCs therapy group (*n* = 6)/Post-MSCs therapy group (*n* = 6)	1.0 × 10^8^	6 months	Significantly improved liver function, METAVIR score, Child–Pugh score, MELD score, and quality of life for patients with liver cirrhosis	Huang et al. [53]
Alcoholic cirrhosis	BM-MSCs	Hepatic artery	Pre-MSCs therapy group (*n* = 11)/Post-MSCs therapy group (*n* = 11)	5.0 × 10^7^/kg	12 weeks	Improved histological features and Child–Pugh score, decreased transforming growth factor-β1, type 1 collagen and α-smooth muscle actin level	Jang et al. [54]
Alcoholic cirrhosis	BM-MSCs	Hepatic artery	Control group (*n* = 18)/One-time MSCs group (*n* = 18)/Two-time MSCs group (*n* = 19)	5.0 × 10^7^/kg	12 months	Improved tissue fibrosis (reduction in the proportion of collagen) and liver function (improvement of the Child–Pugh scores)	Suk et al. [55]
HCV-positive patients with end-stage liver disease	BM-MSCs	Peripheral vein	MSCs transplantation group (*n* = 20)/Control group (*n* = 20)	1.0 × 10^6^/kg	26 weeks	Improved liver synthetic functions (improved ALT, AST and S-albumin levels, as well as the Child–Pugh score and performance score) and hepatic fibrosis (decreased the serum levels of the hepatic fibrosis markers, PIIICP and PIIINP)	Salama et al. [56]
Primary biliary cirrhosis with a suboptimal response to UDCA treatment	UC-MSCs	Peripheral vein	Primary biliary cirrhosis patients with a suboptimal response to UDCA treatment (*n* = 7)	0.5 × 10^6^/kg	48 weeks	Improved liver function (serum alkaline phosphatase and γ-glutamyltransferase levels decreased) and clinical symptoms (fatigue and pruritus were alleviated)	Wang et al. [57]
Liver cirrhosis caused by autoimmune diseases	BM-MSCs, UC-MSCs and cord blood-MSCs (CB-MSCs)	Peripheral vein	UC-MSCs transplantation group (*n* = 23)/ CB-MSCs transplantation group (*n* = 2)/BM-MSCs transplantation group (*n* = 1)	1.0 × 10^6^/kg	2 years	Reduced the mean total bilirubin and prothrombin time, improved the average serum albumin levels and MELD score, without serious adverse events	Liang et al. [58]
Decompensated liver cirrhosis after splenectomy	Autologous bone marrow	Portal vein	Autologous bone marrow transfusion group (*n* = 15)/Control group (*n* = 10)	1.0–2.0 × 10^6^/kg	6 months	Improved liver function (improved ALB, ALT, and cholinesterase levels and decreased liver stiffness measurement and AFP)	Zhang et al. [59]
ACLF	BM-MSCs	Peripheral vein	Stem cell group (*n* = 4)/Placebo group (*n* = 5)	1.0 × 10^6^/kg	90 days	Improved Child–Pugh score, MELD, and ACLF classification	Schacher et al. [60]
HBV-related ACLF	BM-MSCs	Peripheral vein	Standard medical therapy group (*n* = 54)/MSCs transplantation group (*n* = 56)	1.0–10 × 10^5^/kg	24 weeks	Improved liver function, decreased the incidence of severe infection, and significantly improved the 24-week survival rate	Lin et al. [61]
HBV-related ACLF	UC-MSCs	Cubital vein	MSCs transplantation group (*n* = 24)/Control group (*n* = 19)	0.5 × 10^6^/kg	72 weeks	Partially improved liver function (improve serum total bilirubin and Model for End-Stage Liver Disease scores), reduced the incidence of severe infections (reduce the incidence of severe infection, and the mortality of multiple organ failure and severe infection), and reduced patient mortality	Shi et al. [62]
Preparing for liver transplantation	BM-MSCs	Peripheral vein	MSCs transplantation group (*n* = 10)/Control group (*n* = 10)	1.0–2.0 × 10^6^/kg	12 months	Induced mild positive changes of immunoregulatory T cells and NK cells in peripheral blood	Casiraghi et al. [63]
Decompensated cirrhosis	BM-MSCs	Peripheral vein	MSCs transplantation group (*n* = 15)/Placebo group (*n* = 12)	1.95 × 10^8^	12 months	No beneficial effect (no effect on the Child–Pugh score, MELD-Na score, serum albumin, INR, serum transaminases and liver volume)	Mohamadnejad et al. [64]
HBV-induced liver failure	BM-MSCs	Hepatic artery	MSCs transplantation group (*n* = 53)/Control group (*n* = 105)	Not mentioned	192 weeks	Improved the short-term curative effect (improve the levels of ALB, total bilirubin, and prothrombin time and MELD score), and not improve the long-term curative effect (no effect on the incidence of HCC and mortality)	Peng et al. [65]
Liver cirrhosis	BM-MSCs	Peripheral vein	Pre-MSCs therapy group (*n* = 25)/Post-MSCs therapy group (*n* = 12)	1.0 × 10^6^/kg	6 months	MSCs could not reach the liver in a sufficient amount	Kantarcıoğlu et al. [66]

A phase I–II randomized clinical trial was performed to evaluate the initial efficacy and safety of BM-MSCs in patients with stage 2 and 3 ACLF [60]. The results showed that the Child–Pugh score, MELD score, and ACLF score significantly improved in patients who completed the entire MSC infusion regimen. Lin et al. enrolled 110 patients with HBV-associated ACLF treated with a weekly infusion of 1.0–10 × 10^5^ cells/kg for 4 consecutive weeks [61]. At 24-week follow-up, the cumulative survival rate of the MSC group was significantly higher than that of the standard medical therapy group, whereas the incidence of serious infection and mortality of multiple organ failure was much lower in the MSC group than in the standard medical therapy group. Consistent results were obtained in the phase I/II trial performed by Shi et al. [62]. Moreover, Casiraghi et al. conducted an open-label and randomized phase Ib/IIa clinical trial, which supported the safety of infusing MSCs before transplantation in liver transplant recipients and induced slight positive changes in immunoregulatory T cells and NK cells in the peripheral blood [63]. These studies demonstrated that MSC therapy is safe for use in patients with liver disease.

However, a randomized placebo-controlled trial revealed no significant differences in the absolute changes in the Child–Pugh score, MELD-Na score, serum ALB, international normalized ratio, serum transaminase, and liver volume between the MSC and placebo groups during 12-month follow-up [64]. In addition, Peng et al. found that using autologous BM-MSCs to treat patients with chronic hepatitis B-related liver failure did not significantly improve long-term outcomes [65]. However, the study only involved a small number of patients. Further randomized controlled trials are urgently needed to evaluate larger numbers of patients to confirm the efficacy of MSCs in treating liver diseases. The low rate of hepatocyte-like cell transdifferentiation and survival of autologous MSCs in vivo has attracted widespread attention. Shi et al. found that after 7 days of infusion of BM-MSCs through the portal vein of a porcine model of fulminant liver failure, human hepatocytes accounted for only 4.5% of porcine hepatocytes [67]. Notably, because of the poor homing ability of MSCs, effective numbers of these cells cannot be reached in the liver, which may also lead to their poor curative effect. Kantarcıoğlu et al. performed a series of liver biopsies in patients with cirrhosis transplanted with BM-MSCs, which indicated that sufficient numbers of BM-MSCs did not reach the liver [66]. Next, we focus on the mechanism and how to improve the homing of MSCs to enhance their therapeutic effect in liver disease.

## Mechanisms of MSC homing

**Fig. 2:**
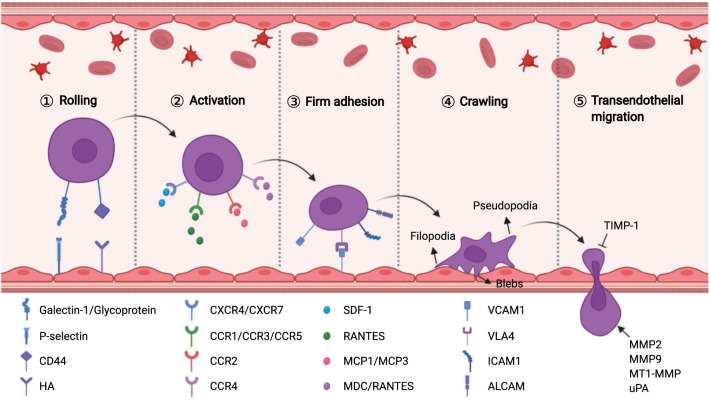
The homing mechanism of MSCs. Schematic summarizing the molecular mechanisms facilitating each step of MSC homing

MSCs can home to sites of damaged tissue, which is the premise of their application in the treatment of systemic diseases [68]. The homing capability of MSCs was first proposed in 2002 by Saito et al. [69]. Subsequently, considerable evidence from numerous studies indicated that exogenous MSCs transplanted into the human body were preferentially captured by the vascular system of the target tissue and then migrated to the target tissue across vascular endothelial cells, which was similar to cells equipped with "GPS" [70]. Particularly, ischemic-damaged tissues can attract MSCs that can home to damaged tissues where they play a therapeutic role. However, unlike the process of leukocyte migration to inflammatory sites, the mechanism of MSC homing is not well understood. In general, MSC homing can be divided into non-systemic homing and systematic homing [71]. Non-systemic homing refers to local transplantation of MSCs to the injured site. Systemic homing of MSCs is guided by homing-promoting factors released from damaged or inflamed tissues, which is similar to the migration of circulating leukocytes to inflammatory sites and is categorized into five consecutive steps: (1) rolling, (2) activation, (3) firm adhesion, (4) crawling, and (5) transendothelial migration (Fig. 2).

### Rolling

Upon activation of endothelial cells, the interaction between upregulated P-selectin, L-selectin, E-selectin, P-selectin glycoprotein ligand 1, E-selectin ligand or s-Lex expressed on leukocytes mediates the binding and rolling of leukocytes, which is a prerequisite for cell migration [72]. Interestingly, Rüster et al. found that MSCs bind to endothelial cells in a P-selectin-dependent manner [73]. However, unlike circulating leukocytes or hematopoietic progenitor cells, MSCs do not express P-selectin ligands, such as P-selectin glycoprotein ligand 1 and CD24, indicating that other ligands interact with P-selectin on the MSC surface. In fact, glycoproteins and galectin-1 expressed on MSCs have been identified as alternative P-selectin ligands. Because of the low affinity between P-selectin and glycolipid or glycoprotein-specific oligosaccharide chains on the surface of MSCs and influence of blood flow velocity, MSCs adhere, separate, re-adhere, and re-separate in blood vessels, showing rolling motion. Additionally, a previous study suggested that MSC homing decreases significantly when its receptor (CD44) is blocked using anti-CD44 antibodies [74]. The CD44 receptor is a ubiquitous transmembrane glycoprotein, also known as a homing receptor, which mediates the homing of leukocytes or hematopoietic stem cells by binding to E-selectin [75]. However, blocking the expression of E-selectin on endothelial cell surfaces does not affect MSC homing, indicating that E-selectin is not the binding site of CD44. Many studies have identified hyaluronic acid (HA) as the potential binding site of the CD44 receptor to mediate MSC homing [74].

### Activation

Chemokines secreted by vascular endothelial cells or damaged tissues that interact with receptors expressed on MSCs can trigger activation of integrin adhesiveness and ultimately mediate MSC migration. Once the chemokine binds to its receptor, the downstream signals talin and kindlin can move to the cell membrane and bind to the integrin β tail, resulting in a transition from a low-affinity to high-affinity conformation, which is crucial for cell adhesion, migration, and assembly of ECM [76]. Stromal cell-derived factor-1 (SDF-1) is a small chemokine in the CXC chemokine family and plays a key role in MSC transportation and homing [77]. Normal blood vessels and tissues typically do not express SDF-1 or may express them in small amounts; following damage, the expression of SDF-1 is upregulated. SDF-1 binds to MSC-expressed C-X-C-motif chemokine receptor 4 (CXCR4) and induces MSCs to mobilize and home to damaged tissues along a concentration gradient of SDF-1 to exert therapeutic effects. Ling et al. found that in a thioacetamide-induced liver injury model, SDF-1 was highly expressed in the liver tissues and promoted MSC homing to the injury site [78]. However, migration of MSCs to the injured liver was partially blocked by AMD3100 or anti-CXCR4 antibodies. Moreover, CXCR7 has also been identified as a receptor of SDF-1, which is involved in MSC homing. Other chemokines and receptors are also activated during MSC homing, such as MCP1/CCR2, MCP3/CCR2, MDC/CCR4, and RANTES/CCR1, 3, 4, and 5 [71]. The MSCs also express multiple receptors, such as CCR10, CXCR5, and CXCR6, although their roles remain to be explored.

### Firm adhesion

After entering the peripheral blood circulation, MSCs continuously roll along with vascular endothelial cells through the action of blood flow and low affinity of selectins. Adhesion of MSCs to the endothelium is mediated by the activation of integrins, which are activated during the interaction between chemokines and their receptors. Numerous studies have revealed that very late antigen 4/vascular cell adhesion molecule 1 (VLA4/VCAM1) plays a key role in the firm adhesion between MSCs and endothelial cells [73]. MSC expresses VLA4 (also known as integrin α4β1), which is activated in response to chemokines such as SDF-1. After activation, VLA4 integrin binds to VCAM1 in endothelial cells [79]. Subsequently, the signaling pathway of cell adhesion is activated, which promotes tight adhesion between MSCs and vascular endothelial cells. Interestingly, MSCs themselves also express the adhesion molecules VCAM1 (CD106), intercellular adhesion molecule 1 (ICAM1, also known as CD54), intercellular adhesion molecule 3 (ICIM3, also known as CD50), and activated leukocyte cell adhesion molecule (ALCAM, also called CD166) [80, 81].

### Crawling

After establishing firm endothelial adhesion, MSCs crawl along the inner wall of blood vessels under the chemotactic gradient and search for the optimal location for transendothelial migration [82]. Endothelial crawling involves filopodia, pseudopodia, and cell polarization. Formation of the intracellular linker molecules FROUNT and CCR2 clusters is crucial for MSC polarization. Activation of the CCR2/FROUNT/PI3K signaling pathway can facilitate the formation of actin filaments and pseudopodia and then mediate cytoskeleton reorganization [83]. Notably, MSCs exhibit nonapoptotic membrane blebbing activity during crawling, particularly when they are in close contact with the endothelium [84]. This is similar to the previously described activity of metastatic tumors and embryonic germ cells but differs from the lamellipodia and invadosomes formed during the migration of leukocytes across the endothelium [84, 85].

### Transendothelial migration

Any transmigrating cell must overcome the barriers of the endothelial cell layer, basement membrane, and pericyte sheath to complete transendothelial migration [86]. To accomplish this, MSCs destroy the endothelial basement membrane by secreting MMPs, which can degrade the major components of the endothelial basement membrane (such as type IV collagen and laminin). Studies have confirmed that MMP2, MMP9, and MT1-MMP positively affect the cross-endothelial migration of MSCs [87, 88]. The maturation and enzymatic activity of MMPs are modulated by various proteins, the most important of which are TIMPs [89, 90]. Ries et al. showed that silencing of MMP2 or MT1-MMP reduced MSC migration, whereas TIMP-1 knockdown had the opposite effect [91]. In addition, urokinase-type plasminogen activator (uPA) has been found in prominent pseudopodia of MSCs [92]. uPA is a proteolytic enzyme that mediates the proteolytic cleavage of plasminogen to produce plasmin, which decomposes components of the ECM (such as fibrin, laminin, or type IV collagen) [93]. The uPA activity is related to the invasive ability of leukocytes, endothelial cells, and metastatic tumor cells. Krstić et al. confirmed that uPA enhanced MSC migration in an ERK1- or MAPK-dependent manner [94].

## Strategies to promote MSC homing in the treatment of liver diseases

For MSCs to exert their multiple biological functions, sufficient viable cell quantities are needed to reach the damaged tissue, which is the basis of MSC treatment. However, transferring MSCs to the site of damage or functional loss is difficult. This may be partly related to the loss of stemness after over-passaging of MSCs. The MSCs gradually lose or decrease the expression of homing molecules (such as CXCR4) during amplification in vitro [95]. The aging of MSCs in vitro and the accumulation of intercellular oxidative damage also affect cell proliferation and homing rates [96]. The homing rate of MSCs transplanted via different routes also differs [97]. In addition, most MSCs are found to be trapped in the lungs after peripheral vein transplantation. This can be attributed to the mechanical impedance of the capillary system [98]. Nowdays, various strategies have been adopted to increase the homing rate of MSCs to improve their efficacy in treating liver diseases.

### Administration routes of MSCs

**Fig. 3:**
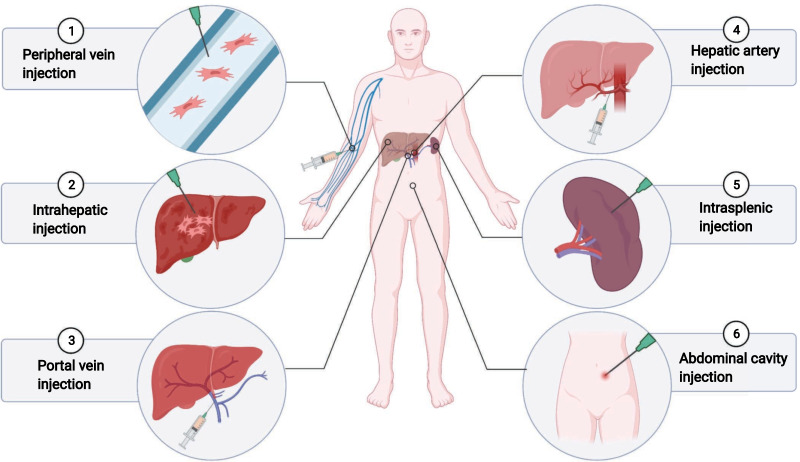
Various routes of MSC transplantation in liver disease. Overview of the routes of MSC injection in animal experiments and clinical trials

Selection of the transplantation route may directly affect the number of engrafting cells colonizing the liver, which in turn affects the therapeutic effect. Perhaps the most immediate improvement method would be the infusion of MSCs at or near the liver (non-systemic homing) rather than traditional intravenous infusion (systemic homing). Currently, clinical methods for cell transplantation mainly include hepatic artery infusion, portal vein transplantation, intrahepatic injection, intrasplenic injection, peripheral vein transplantation, and intraperitoneal injection (Fig. 3). Although research has confirmed that different routes of MSC transplantation can cure different liver injuries, there are few comparative studies of these routes of MSC transplantation [99]. Sang et al. compared the effects of MSC transplantation in the treatment of acute liver failure (ALF) in the peripheral vein, portal vein, hepatic artery, and intraperitoneal cavity [100]. The results showed that portal vein transplantation of MSCs is preferred over other transplantation approaches because it significantly improved liver function, inhibited apoptosis, and prolonged survival. Sun et al. also compared the effectiveness of four BM-MSC transplantation routes portal vein, hepatic artery, tail vein, and intraperitoneal injection for treating ALF. However, the choice of blood vessels in the implantation route does not impact the therapeutic effect, except the intraperitoneal transplantation of MSCs exhibits no therapeutic effect [101]. Intrahepatic injection appears to be an ideal route for transplanting MSCs, as it can effectively reduce the number of cells stranded in circulation. It was found that animals injected intraperitoneally showed that the hepatocytes derived from MSCs preferentially distributed around the portal vein, whereas the intrahepatic injection resulted in extensive distribution throughout the liver parenchyma [102]. Some researchers have also suggested that hepatic artery injection is the best route of infusion and shows a better homing effect [103]. In addition, vascular patency may be an important factor in the successful homing of MSCs to target tissues. Yukawa et al. found that combined use of heparin and MSCs significantly reduced the accumulation of AD-MSCs in the lung and effectively increased the accumulation of transplanted AD-MSCs in the liver [104]. However, current research on MSC transplantation pathways has some potential limitations. The optimal application routes of MSCs in treating liver diseases requires further exploration, and the relevant mechanisms are not fully understood.

### Pretreating MSCs and optimizing cultivation conditions

Since MSCs lose or downregulate the expression of homing molecules during in vitro amplification, researchers have attempted to improve the homing of MSCs by pretreating the cells or optimizing the culture conditions for the treatment of liver disease. The UC-MSCs pretreated with rapamycin can enhance the homing and migration ability of these cells by enhancing immunosuppression and enhancing CXCR4 expression, thereby enhancing the protection against liver ischemia/reperfusion injury [105]. Melatonin pretreatment can also improve BM-MSCs homing by downregulating the expression of TGF-β1 and Bax, while upregulating MMPs and BCL2 expression, and reduce the accumulation of collagen and lipids in liver fibrosis [106, 107]. The intravenous anesthetics dexmedetomidine and midazolam or heat shock pretreatment can also improve the migration ability of MSCs and increase the number of MSCs that home to ischemia/reperfusion injured tissues, thereby significantly improving liver function [108, 109]. Hajinejad et al. found that resveratrol significantly promotes the expression of AKTs and CXCL12 (SDF-1) in the cirrhotic liver, and SDF-1α pretreatment can increase CXCR4 and MMP9 levels in BM-MSCs, both of which can significantly promote the homing of MSCs to liver tissues and reduce their accumulation in the lungs and spleen [110]. The HGF pretreatment can promote MSC homing to the damaged liver by upregulating the expression of c-Met and phosphorylated Met in MSCs, thereby mediating MSC-induced liver repair [111]. IL-1β pretreatment can enhance MSC homing, at least partially, by increasing the expression of CXCR4 and further improving the efficacy of MSCs in ALF [112]. Pretreatment of AD-MSCs with eugenol or NO enhances their homing and proliferation abilities and their ability to treat liver fibrosis in rats [113, 114]. However, although hypoxia pretreatment of MSCs has been shown to enhance the therapeutic effect of liver disease treatment, studies are needed to explore whether hypoxia preconditioned MSCs can improve the homing rate of the damaged liver in liver disease.

The MSCs inevitably undergo rapid aging during amplification, significantly affecting their homing and paracrine functions. Choi et al. found that third-generation MSCs grew at the fastest rate and then gradually declined [115]. Cytokine secretion decreased gradually during prolonged culture, with the most significant decrease observed at passages 7 and 9. The gradual decrease in IL-6 and VEGF expression appeared to be associated with a decreased growth rate during culture. Moghadam et al. also observed reduced expression of VCAM1 and IL-6 in BM-MSCs during subculture [116]. In AD-MSCs, the mRNA levels of IL-10 were reduced in later generations compared to in the 3rd passages. Therefore, the long-term culture of MSCs may progressively lead to a loss of proliferative capacity and differentiation potential, and early passage MSCs exhibiting stability and more effective anti-inflammatory properties are likely to have beneficial effects in patients. In addition, the culture density likely affects the migration ability of MSCs. Becker et al. found that high culture fusion increased the production of TIMP-3 and reduced the cross-endothelial migration of MSCs [117]. However, Kim et al. found that proliferation-associated genes were highly expressed in low-density MSCs, whereas high-density (approximately 90% confluency) MSCs highly expressed several cytokines, chemokines, and growth factor-related genes participate in immunosuppression, migration, and reconstruction of damaged tissues [118]. Therefore, it is unclear whether the optimal density for MSC culture is appropriate for therapeutic applications. Co-cultivation with other cells also affects the migration ability of MSCs. Ran et al. co-cultured amniotic membrane-derived MSCs (AMSCs) with amniotic epithelial cells and observed upregulated CXCR4 on the surface of AMSCs and an enhanced in vitro migration capacity of these cells [119]. Activated endothelial cells can improve the differentiation potential and migration activity of MSCs through direct contact or paracrine regulation [120]. In addition, co-culturing MSCs with Sertoli cells upregulates the expression of homing genes such as CXCR4 and MMP2 in MSCs [121, 122]. The above results indicate that either pretreatment or optimization of MSC culture conditions can improve the cell homing effect.

### Gene modification

The homing process of MSCs is mainly mediated by interactions between ligands and receptors. Changing the expression level of receptors/ligands on MSCs is a potential method for improving the homing efficiency within target tissues. Overexpression of HGF and c-Met can effectively promote homing of MSCs to the liver injury site, thereby improving the repair effect of MSCs for treating ALF [123, 124]. Overexpression of CXCR4 increases the mobilization and engraftment of MSCs in liver transplants and improves their effect on hepatocyte proliferation [125]. The homing and colonization rates of VEGF165-MSCs are also increased, leading to significant improvement of liver injury in ALF rats and the promotion of liver regeneration [126]. The BM-MSCs transfected with Akt1 exhibit better homing ability and longer persistence in the damaged liver and show survival advantages and enhanced immune regulatory functions in vivo and in vitro [127]. In addition, overexpression of microRNA-27b can inhibit the directional migration of primary cultured CRCX4-positive MSCs by directly downregulating the expression of SDF-1α [128]. The BM-MSCs overexpressing pigment epithelium-derived factor showed preferential homing to hepatocellular carcinoma in in vivo and in vitro migration tests and significantly inhibited the growth of primary liver tumors and development of lung metastases [129]. The above studies showed that overexpressed receptors or ligands of MSCs can directly promote MSC homing to target tissues by interacting with specific cytokines released from damaged tissues.

### Other strategies for MSC mobilization

The homing ability of MSCs can be improved through cell surface engineering. Liao et al. modified the LSEC-targeting peptide RLTRKRGLK (RK) on the surface of AD-MSCs using a bioorthogonal click reaction [130]. Compared with unmodified AD-MSCs, RK-modified AD-MSCs showed significantly higher liver accumulation, leading to better treatment results. Hwang et al. demonstrated that lipid-coupled heparin-coated AD-MSCs had a higher efficiency of liver-targeted delivery and significantly enhanced liver regeneration and anti-inflammatory effects in mouse ALF models [131].

The local microenvironment of the liver is important for MSC homing. In addition to the methods described above, modification of target tissues can promote MSC homing. Vittorio et al. injected MSCs loaded with carbon nanotubes into the portal vein of rats to explore the effect of the magnetic force exerted by carbon nanotubes on MSC homing [132]. The results showed that carbon nanotubes can guide MSCs to migrate to the magnetic source in vivo and in vitro, increasing their transplantation and homing in the liver tissue. Nasir et al. found that using IL-6 to pretreat fibrotic livers to improve the liver microenvironment significantly promoted the homing of MSCs and reduced fibrosis and apoptosis [133]. Shao et al. irradiated the right liver of cirrhotic tissue (15 Gy) 4 days before transplantation. This preliminary liver irradiation significantly promoted the homing and re-proliferation of BM-MSCs and significantly improved liver fibrosis in rat models [134]. Ultrasound-targeted microbubble destruction therapy effectively induces a favorable microenvironment for cell implantation, thus improving liver homing of BM-MSCs, which may be mediated by upregulation of adhesion molecules and cytokine expression [135]. Sun et al. confirmed that combined application of BM-MSCs-HGF and ultrasound-targeted microbubble destruction technologies further promoted the homing of BM-MSCs and, more importantly, further improved their response to liver fibrosis [136]. External stimuli, such as mechanical stretching, physiological DC electric field, non-invasive pulse-focused ultrasound, and biological scaffolds, can control or induce the direct migration of MSCs. However, whether they can improve the therapeutic efficacy of liver disease treatments by promoting MSC homing requires further investigation.

## Conclusions and future directions

Many studies have shown that MSCs can play a therapeutic role in treating liver diseases through various mechanisms: (1) inhibiting hepatocyte apoptosis and promoting hepatocyte regeneration, (2) para-secretion of a variety of cytokines to synergistically protect against liver fibrosis, and (3) regulating immunity to reduce the inflammatory response and restore the steady state of the body. Multiple clinical trials have verified the therapeutic efficacy of MSCs in liver diseases. However, some clinical studies revealed that only a certain number of infused MSCs home to the liver, and MSC transplantation has no significant benefit on the long-term prognosis of patients. Therefore, in-depth studies are underway to identify relevant mechanisms of MSC homing and explore novel strategies for improving the efficacy of MSC therapies.

Improving the homing ability of MSCs may be key to their therapeutic effects. MSC homing is a multi-step process mediated by specific molecular interactions. Although homing has been studied since the 1970s, many aspects of this process remain unknown and require further confirmation. Numerous studies have focused on promoting the homing ability of MSCs to improve the efficacy of the treatment for liver diseases, mainly from the four following four perspectives. (1) Transplantation route: the choice of MSC transplantation route may directly affect the colonization of transplanted cells in the liver. Peripheral intravenous infusion of MSCs is easy, economical, and can perform multiple times; however, the proportion of cell colonization to the liver is low, making this approach suitable for allogeneic MSCs. The portal vein route may aggravate portal hypertension, resulting in a risk of hemorrhage. Therefore, portal vein injection may be more suitable for patients with no risk of portal hypertension. Non-systematic homing of MSCs can also be achieved by liver or spleen puncture injection; however, the amount of each injection is limited, and patients with liver cirrhosis are at risk of bleeding and liver rupture. Hepatic artery injection is commonly performed for autologous MSC transplantation, and MSCs can be injected directly into the liver, although this method is not suitable for multiple treatments. There are still limitations in research on MSC transplantation routes. It may be necessary to consider the specific conditions of patients with liver disease to further evaluate the most suitable administration route in different types of patients. (2) Pretreatment or optimization of MSC culture conditions: pretreatment or optimization of culture conditions before an application is an effective method for improving the therapeutic effects of MSCs. Although pretreatment or optimization of culture conditions can successfully improve the therapeutic efficacy of MSCs in treating liver diseases, it can also affect the phenotypic and paracrine functions of MSCs, and thus, further exploration is required before their future clinical application. (3) Modifying MSCs to enhance their homing: modifying MSCs through gene editing or chemical modification is an active area of research; however, these methods may cause biosafety issues, and several preclinical studies are required to explore their safety and effectiveness. (4) Modifying target tissues: modifying target tissues to make them more attractive appears to be a promising approach. Studies have confirmed that injecting homing factors into target tissues, genetic modification of target tissues, radiation, or ultrasound technology enhances MSC homing, but there are few studies on liver disease. In addition, bioactive scaffolds that deliver cytokines can act as “homing signals” for MSCs but may be difficult to further optimize and exhibit safety and cost issues, and there are limitations to their clinical application for treating liver disease [81].

Although poor homing may be a major limitation to implementing MSC-based therapies, other factors also affect their application potential. Currently, unified specifications for the clinical application of MSCs are lacking. The cell source, dose, route, optimal time of infusion, and curative effect have shown some inconsistent results in various clinical trials. Freshly isolated MSCs have strong homing efficiency and tissue repair effects, whereas MSCs used in clinical experiments are typically cryopreserved, which damages the homing ability of MSCs and shortens their durability in vivo and tissue repair. MSCs derived from different tissues sources have different biological characteristics and differentiation abilities. The affinity between MSCs from different sources and target tissue should also be taken into consideration. Therefore, standardized methods for using MSC treatment for liver diseases are needed. In addition, MSC therapy is associated with risks of iatrogenic neoplasia, cellular embolism, and thrombosis. However, the safety of MSC transplantation in the case of malignant tumors requires further analysis, as MSCs home to tumor sites through a similar homing mechanism. Up to now, the Food and Drug Administration (FDA) or European Medicines Administration (EMA) has approved a variety of MSC preparations (such as Osteocel, Stemirac, and Alofisel) to treat numerous diseases. However, it is worth noting that among the marketed MSC preparations, Prochymal, the world's first stem cell drug, is priced at US$ 200,000 per treatment course, Stemirac is priced at about $ 135,000 per session and Temcell is priced at about $ 120,000 per session. Because the government and insurance companies cannot be persuaded to reimburse their expenses, the delisting or suspension of stem cell products, such as Chondrocelect, in the European Union has received considerable attention. Therefore, the pharmacoeconomics of MSC preparations remains a key topic in current stem cell research.

In conclusion, we described the application prospects of MSCs for treating liver diseases. However, further studies are needed to investigate the homing mechanism of MSCs and various strategies for improving homing.

## Data Availability

Not applicable.

## Abbreviations

MSC: Mesenchymal stem cell
ALB: Human albumin
AFP: α-Fetoprotein
UC: Umbilical cord
AD: Adipose-derived
BM: Bone marrow
hP: Human placenta
DCs: Dendritic cells
NK: Natural killer
HGF: Hepatocyte growth factor
EGF: Epidermal growth factor
FGF: Fibroblast growth factor
OSM: Oncostatin M
Dexa: Dexamethasone
ITS: Insulin transferrin selenium
PGE2: Prostaglandin E2
IDO: Indoleamine-2, 3-dioxygenase
NO: Nitric oxide
HLA-G: Human leucocyte antigen-G
Gal: Galectin
CCL2: CC chemokine ligand 2
HO-1: Heme oxygenase-1
IL1Ra: Interleukin 1 receptor antagonist
HSCs: Hepatic stellate cells
ECM: Extracellular matrix
MMPs: Matrix metalloproteinases
TIMP: Metalloproteinase tissue inhibitor
TSG-6: Tumor necrosis factor α stimulated gene-6
MFGE-8: Milk fat globe-epidermal growth factor-8
ACLF: Acute-on-chronic liver failure
MELD: Model for end-stage liver disease
HA: Hyaluronic acid
SDF-1: Stromal cell-derived factor-1
CXCR: C-X-C-motif chemokine receptor
MCP: Monocyte chemotactic protein
CCR: C–C motif chemokine receptor
MDC: Mevalonate diphosphate decarboxylase 2
RANTES: Recombinant human rantes
VLA4: Very late antigen 4
VCAM1: Vascular cell adhesion molecule 1
ICAM1: Intercellular adhesion molecule 1
ICIM3: Intercellular adhesion molecule 3
ALCAM: Activated leukocyte cell adhesion molecule
PI3K: Phosphatidylinositol 3-kinase
MT1-MMP: Membrane type 1-matrix metalloproteinase
uPA: Urokinase-type plasminogen activator
ERK1: Extracellular signal-regulated kinase 1
MAPK: Mitogen-activated kinase-like protein
BCL2: B cell lymphoma 2
VEGF: Vascular endothelial growth factor
